# Identifying key genes associated with recurrence in non-small cell lung cancer through TCGA and single-cell analysis

**DOI:** 10.3389/fmed.2025.1549969

**Published:** 2025-06-11

**Authors:** Weiyuan Li, Duo Han, Chunxiao Cao, Yuning Xie, Jingxia Shen

**Affiliations:** ^1^North China University of Science and Technology Affiliated Hospital, Tangshan, Hebei, China; ^2^Zunhua People’s Hospital, Tangshan, Hebei, China; ^3^School of Public Health, North China University of Science and Technology, Tangshan, Hebei, China

**Keywords:** NSCLC, TCGA, WGCNA analysis, Single-Cell Analysis, KCNA7, FOX NSCLC, fox

## Abstract

**Objective:**

This study aims to mine the TCGA database for differentially expressed genes in recurrent lung cancer tissues, determine the relationship between these recurrent genes and lung cancer at the single-cell level, and identify potential targets for lung cancer treatment.

**Methods:**

Data for lung adenocarcinoma (LUAD) and lung squamous cell carcinoma (LUSC) were obtained from the TCGA database and grouped based on clinical recurrence information. Single-cell data from GSE131907 were downloaded from the GEO database. R was utilized to screen for differentially expressed genes (DEGs), followed by weighted gene co-expression network analysis (WGCNA) of these DEGs. Additionally, the GSEA database was employed to visualize differential pathways and identify key genes. The relationship between the expression of these key genes and lung cancer recurrence was validated using the GSE131907 single-cell dataset.

**Results:**

A total of 2,239 differentially expressed genes were identified in the LUAD dataset, while 3,404 differentially expressed genes were found in the LUSC dataset. WGCNA revealed that the lapis lazuli module gene set was associated with recurrence. Validation at the single-cell level indicated that the FOXI1, FOXB1, and KCNA7 genes were linked to lung cancer progression.

**Conclusion:**

The differentially expressed genes primarily influence NSCLC recurrence through involvement in biological processes related to metabolism and hormone secretion pathways. Notably, the KCNA7 and FOX gene families were identified as critical for NSCLC recurrence. This study highlights specific genes within proliferation and cell cycle pathways as key therapeutic targets for managing NSCLC recurrence.

## 1 Introduction

Lung cancer is the leading cause of cancer-related mortality worldwide (1). It is a malignant tumor that originates from alveolar and bronchial epithelial cells and is histologically classified into two primary subtypes: small cell lung cancer (SCLC) and non-small cell lung cancer (NSCLC). Among these, NSCLC constitutes approximately 85% of lung cancer cases, predominantly manifesting as lung adenocarcinoma (LUAD) and lung squamous cell carcinoma (LUSC). NSCLC cells possess self-renewal capabilities, enabling continuous proliferation and destruction of surrounding tissues, as well as the release of tumor-associated factors, which contribute to high rates of recurrence and metastasis. This significantly impacts patients’ quality of life and survival (2). The progression of lung cancer lesions involves complex interactions among various genetic factors that may exhibit aberrant expression within a regulatory network (3, 4). With advancements in high-throughput technologies, genome-wide gene expression microarrays have become instrumental in identifying novel biomarkers for cancer research (5). RNA sequencing (RNA-seq) is an efficient high-throughput technique that quantifies transcripts, identifies new transcription units, and detects differentially expressed genes (DEGs) across samples. When combined with bioinformatics approaches, RNA-seq facilitates the characterization of lung cancer prognosis, enhances understanding of molecular biology, identifies prognostic markers, and contributes to therapeutic drug design (6, 7). The Cancer Genome Atlas (TCGA) serves as the largest repository of sequencing data, providing comprehensive information on tumor stage, metastasis, survival outcomes, patient demographics, and corresponding clinical diagnostic and follow-up details (5). The Gene Expression Omnibus (GEO) database, curated by the International Centre for Biotechnology Information (NCBI), is one of the most extensive gene expression repositories globally (8). Utilizing bioinformatics methods, we aimed to download prognostic information and sequencing data for various lung cancer subtypes from GEO and TCGA databases, subsequently identifying abnormally expressed genes associated with NSCLC prognosis.

Weighted gene co-expression network analysis (WGCNA) effectively utilizes gene expression data to categorize genes into distinct modules with similar expression profiles, transcending traditional analyses focused solely on individual highly or poorly expressed genes. This modular approach, grounded in biological networks, aids in identifying crucial gene modules linked to specific sample traits (9, 10). WGCNA was employed to analyze data and explore gene modules closely associated with NSCLC, partially addressing limitations inherent in differential gene expression analyses and narrowing the scope for candidate cancer marker identification.

A review of literature concerning NSCLC prognostic models reveals a predominance of models emphasizing immunological, metabolic, and other factors (11). However, current studies often neglect the integration of single-cell RNA sequencing (scRNA-seq) data, overlooking the implications of cellular heterogeneity. Furthermore, many prognostic models rely heavily on predicting genes while disregarding other significant predictive variables, such as clinical characteristics including age, gender, and tumor stage. This oversight can lead to inefficiencies in prognostic model development. Recent advancements in sequencing technology have seen scRNA-seq widely adopted as an innovative method for investigating the transcriptomes of diverse cell types (12). Additionally, scRNA-seq elucidates heterogeneity and distinct subpopulations within tumors, quantitatively assessing immune cell infiltration in both normal and tumor tissues—a critical factor influencing treatment response and prognosis in NSCLC (13–16).

In this study, we aim to integrate and analyze publicly available RNA-seq data related to NSCLC. Through WGCNA, we will identify gene modules that are closely related to NSCLC, followed by Gene Ontology (GO) and Kyoto Encyclopedia of Genes and Genomes (KEGG) enrichment analyses of these genes, thereby enhancing our understanding of the underlying molecular mechanisms of NSCLC. By combining WGCNA with differential gene expression analysis, we will isolate DEGs from these modules that are likely critical to NSCLC development, using the expression data of these key genes to construct prognostic models.

## 2 Materials and methods

### 2.1 Data extraction and variance analysis

To comprehensively investigate differential gene expression between recurrent and non-recurrent lung cancer cases, clinical and transcriptomic data for lung adenocarcinoma (LUAD) and lung squamous cell carcinoma (LUSC) were retrieved from The Cancer Genome Atlas (TCGA), with tumor samples stratified by recurrence status based on clinical follow-up records. Raw RNA-seq counts were preprocessed by log2 transformation and normalized for library size and compositional bias using the edgeR package (v4.0.2), followed by filtering to exclude genes with zero expression across all samples. Differentially expressed genes (DEGs) were identified through a generalized linear model (GLM) approach in edgeR, applying stringent thresholds of |log2 fold change (FC)| > 0.5 (indicating a ≥ 1.4-fold change) and an FDR-adjusted *p* < 0.05 (Benjamini-Hochberg method) to ensure robust results. To visualize the findings, hierarchically clustered heatmaps were generated using the pheatmap package, displaying Z-score-normalized expression of top DEGs alongside clinical annotations, while volcano plots (ggplot2) highlighted the statistical significance and magnitude of expression changes, with key DEGs labeled for further investigation. All analyses were conducted in R (v4.2.1), with scripts archived for reproducibility.

### 2.2 Enrichment analysis

Gene function enrichment is crucial for translating high-throughput molecular results into biological significance (17). To elucidate the biological relevance of the identified differentially expressed genes (DEGs), comprehensive functional enrichment analyses were performed in R software. Gene annotation was first conducted using org.Hs.eg.db (v 3.18.0) to map Ensembl IDs to standardized gene symbols. Subsequently, Gene Ontology (GO) enrichment analysis (covering biological processes, molecular functions, and cellular components) and Kyoto Encyclopedia of Genes and Genomes (KEGG) pathway analysis were carried out via the clusterProfiler package (v 4.10.0), with significance thresholds set at an adjusted *p* < 0.05 (FDR correction) and an enrichment score cutoff of *q* < 0.2. For a systems-level perspective, Gene Set Enrichment Analysis (GSEA) was analyzed and visualization using the GseaVis package.

### 2.3 WGCNA analysis

Weighted Gene Co-expression Network Analysis (WGCNA) was performed to identify modules of highly correlated genes, summarize interconnections between modules, and assess associations with external sample traits, thereby identifying candidate biomarkers or therapeutic targets (18). To systematically identify co-expressed gene networks associated with lung cancer recurrence, WGCNA was implemented using the WGCNA package (v1.72.1) in R. The analysis began with rigorous data preprocessing, including normalization of expression matrices and removal of outlier samples based on hierarchical clustering and sample network connectivity (Z-score cutoffs). A weighted correlation matrix was constructed across all genes, and an optimal soft-thresholding power (β) was selected (via scale-free topology fit index > 0.85) to transform the matrix into a signed adjacency matrix, balancing network connectivity and biological relevance. This adjacency matrix was further converted into a Topological Overlap Matrix (TOM) to quantify gene-gene interaction strengths, mitigating spurious correlations. Using average linkage hierarchical clustering and a dynamic tree-cutting algorithm (minimum module size set at 30 genes), genes with similar expression patterns were partitioned into co-expression modules, labeled by color. Modules were correlated with clinical traits such as recurrence status and metastasis, and the most significant module (highest absolute Pearson correlation, *p* < 0.05) was identified as the key recurrence-associated module. Overlapping genes between this module and the previously identified DEGs were classified as differential recurrent metastatic genes (DRMGs), representing robust candidates for further validation as biomarkers or therapeutic targets.

### 2.4 Single-cell analysis

To explore the distribution of hub genes across cell populations, we employed single-cell techniques for analysis and visualization (19). To elucidate the cellular distribution and clinical relevance of recurrence-associated hub genes, we performed scRNA-seq analysis using data from the GSE131907 dataset (22 lung cancer samples). Quality control was rigorously applied via the Seurat package (v4.3.0) in R, retaining cells expressing > 300 genes (nFeature_RNA) and excluding those with > 10% mitochondrial gene content (percent.mt) to minimize low-quality or apoptotic cells. The filtered dataset was normalized using SCTransform to correct for technical variance, followed by principal component analysis (PCA) and uniform manifold approximation and projection (UMAP) for dimensionality reduction. Cell clusters were annotated based on canonical markers (e.g., EPCAM for epithelial cells, PTPRC for immune cells). Hub genes identified from WGCNA and DEG analyses were mapped onto these clusters to assess their cell-type-specific expression patterns, with a focus on tumor and microenvironment subsets implicated in recurrence (e.g., malignant epithelial cells, cancer-associated fibroblasts). Differential expression testing (Wilcoxon rank-sum test) was applied within Seurat to validate hub gene enrichment in recurrence-related cell populations (*p* < 0.05). Results were visualized via dot plots, feature plots, and violin plots using Seurat to highlight gene-cell associations, providing spatial context to molecular drivers of recurrence.

### 2.5 Identification and prognostic validation of protein-protein interaction networks and hub genes

To systematically investigate the functional interactions and clinical implications of key recurrence-associated genes, we employed an integrative bioinformatics approach. Protein-protein interaction (PPI) networks were constructed using String database, incorporating physical interactions, co-expression, genetic interactions, and pathway co-membership data to identify functionally related gene clusters and potential novel interactors. For clinical correlation analysis, we leveraged GEPIA2^1^ and UALCAN^2^ databases to comprehensively evaluate the prognostic significance of core genes, analyzing their expression patterns across multiple clinical parameters: (1) survival outcomes (overall and disease-free survival, log-rank test *p* < 0.05), (2) tumor stage progression, (3) smoking status, (4) lymph node metastasis status, and (5) TP53 mutation subgroups. Furthermore, we investigated DNA methylation profiles of core genes using UALCAN’s TCGA methylation data, examining the association between promoter methylation levels and both gene expression patterns and clinical outcomes. This multi-dimensional analysis enabled us to identify clinically relevant molecular signatures with potential prognostic utility in lung cancer recurrence.

### 2.6 Immunohistochemistry

Tissue samples were fixed in 10% formalin and embedded in paraffin wax. Sections of 4 μm thickness were mounted on glass slides. Prior to staining, slides were deparaffinized in xylene and rehydrated through a series of graded alcohols. Antigen retrieval was performed by heating the sections in citrate buffer (pH 6.0) in a microwave for 15 min. Endogenous peroxidase activity was blocked with 3% hydrogen peroxide for 10 min. Non-specific binding was minimized by incubating the sections with 5% bovine serum albumin for 30 min at room temperature. Primary antibodies against KCNA7 (Cat: PA5-145126, Invitrogen) and FOXB1 (Cat: PA5-18170, Invitrogen) were diluted to 1:500 in antibody diluent and applied to the sections overnight at 4°C. After washing with phosphate-buffered saline (PBS), sections were incubated with horseradish peroxidase-conjugated secondary antibodies for 1 hour at room temperature. Immunoreactivity was visualized using a diaminobenzidine (DAB) substrate solution, and sections were counterstained with hematoxylin. Stained sections were examined under a light microscope, and images were captured for further analysis.

## 3 Results

### 3.1 Differential genes in the lung cancer recurrence dataset

**FIGURE 1:**
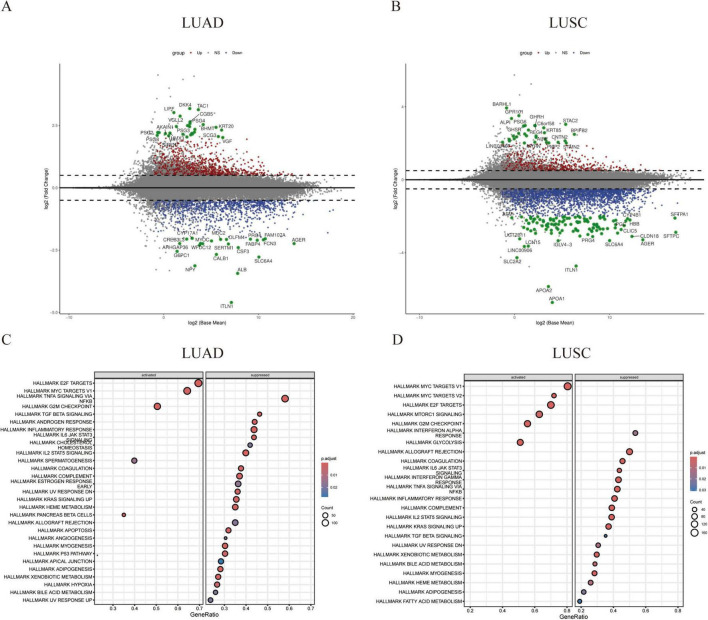
Differences in gene expression profiles between primary and relapsed non-small cell lung cancer. **(A,B)** Volcano plots illustrate the differential gene expression between two patient groups: lung adenocarcinoma (LUAD) and lung squamous cell carcinoma (LUSC). Red and blue points represent differentially expressed genes, while green points denote marker genes within this set. **(C,D)** Gene set enrichment analysis (GSEA) of the comprehensive expression profiles of differentially expressed genes reveals two distinct patterns: upregulated activation and downregulated resistance.

The TCGA lung cancer dataset was analyzed based on recurrence status in lung adenocarcinoma (LUAD) and lung squamous cell carcinoma (LUSC). Differentially expressed genes (DEGs) were identified with thresholds of P < 0.05 and |Log2FC| > 0.5. From the LUAD dataset, we identified 2,239 DEGs, comprising 1,151 up-regulated and 1,088 down-regulated genes. In the LUSC dataset, we found 3,404 DEGs, including 735 up-regulated and 2,669 down-regulated genes (Figures 1A,B). Gene Ontology (GO) term enrichment and Kyoto Encyclopedia of Genes and Genomes (KEGG) pathway analyses were conducted to predict the potential functions of these DEGs in both LUSC and LUAD. Additionally, Gene Set Enrichment Analysis (GSEA) was performed on differential genes from the two datasets to select hallmark gene sets as references. The up-regulated pathways primarily related to cancer cell proliferation included the E2F, MYC, and G2M signaling pathways. Meanwhile, down-regulated pathways were associated with immune response, apoptosis, and necrosis, such as the IL6, TGF-β, TNF, and TP53 signaling pathways (Figures 1C,D). Thus, we conclude that in recurrent non-small cell lung cancer (NSCLC), tumor proliferation is enhanced while immune evasion occurs, with pathways associated with apoptosis and necrosis being down-regulated. This indicates an increase in tumor cell proliferation and metastasis during cancer recurrence, which is also linked to the immune microenvironment.

### 3.2 Weighted co-expression network and key modules

**FIGURE 2:**
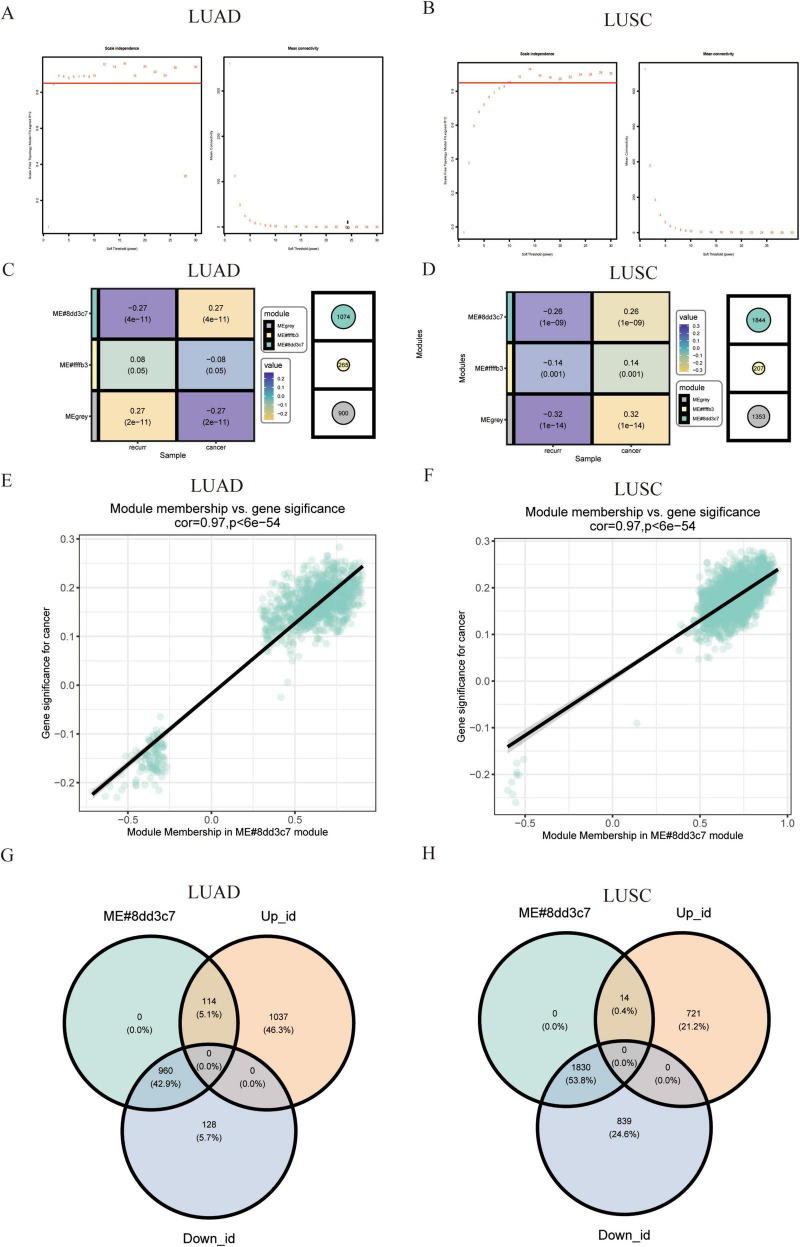
Identification of modules and genes linked to clinical status of lung adenocarcinoma using weighted gene co-expression network analysis (WGCNA). **(A,B)** Scale-free fitting index and average connectivity analysis: this analysis was performed across various soft threshold powers for both LUSC and LUAD. **(C,D)** Heat map of correlation between module characteristic genes and recurrence traits: a heat map visualizes the correlation between module characteristic genes and recurrence traits in both cancers. **(E,F)** Scatter plot of correlation between module characteristic genes and recurrence traits: scatter plots further illustrate the correlation between module characteristic genes and recurrence traits in LUSC and LUAD. **(G,H)** Venn diagram for intersection genes: Venn diagrams identify overlapping genes between differential genes and lapis lazuli modules in LUSC and LUAD, respectively.

To further explore co-expression patterns among the DEGs in lung adenocarcinoma, we performed Weighted Gene Co-expression Network Analysis (WGCNA). We assessed the relationship between DEGs and lung cancer recurrence traits through modular analysis of the two DEG sets from the TCGA NSCLC dataset. To ensure a scale-free network, a minimum soft-threshold power (β-value) of 2 was chosen for both the LUAD and LUSC groups (Figures 2A,B). Two co-expression modules were identified using cluster dendrograms, revealing a strong correlation between the lapis lazuli module and the recurrence trait (Figures 2C,D). Correlation analysis of NSCLC recurrence traits with genes in each module indicated that the lapis lazuli module exhibited the highest correlation coefficient with lung cancer recurrence (correlation coefficient of 0.97, *P* < 0.05) (Figures 2E,F). These results suggest a positive correlation between the lapis lazuli module and the degree of tumor recurrence. Finally, we screened differential genes against module genes, and the intersection of DEGs with the lapis lazuli module is depicted in a Venn diagram (Figures 2G,H).

### 3.3 Single-cell analysis

**FIGURE 3:**
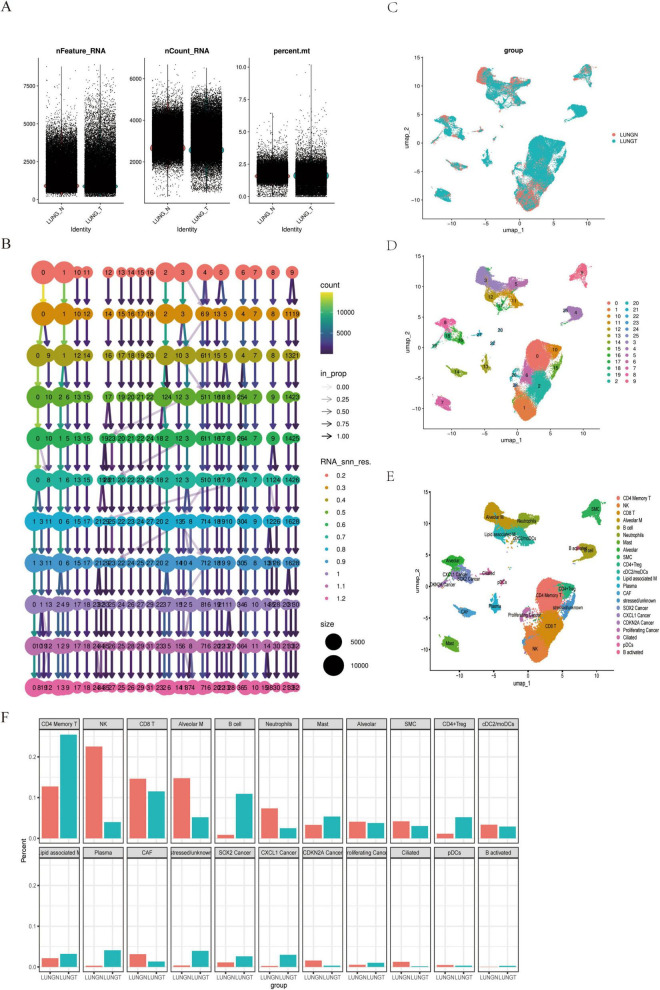
Single-cell analysis of paracancerous and lung cancer tissues. **(A)** QC Violin: illustrates quality control metrics for single-cell sequencing data. **(B)** Bifurcation tree: provides a hierarchical view of cell populations at single-cell resolution. **(C)** UMAP clustering: displays dimensionality reduction clustering for normal and cancer tissues. **(D)** UMAP 26 clusters: shows 26 distinct cell clusters. **(E)** Annotated UMAP: provides labeled clusters for cell type identification. **(F)** Cell subset distribution: depicts the proportional distribution of cell subsets.

To validate the expression levels of the DEGs, we applied the Seurat package to the lung cancer GSE131907 dataset. Quality control was performed based on nFeature_RNA > 300, min.cells = 3, and percent.mt < 10%. Subsequently, dimensionality reduction and clustering were conducted using HARMONY with a resolution of 0.05 (Figures 3A,B). This analysis identified 26 distinct subpopulations (Figure 3C). Automated annotation via SingleR revealed 22 cell subpopulations, including T cells (CD4 Memory T, CD8 T, CD4 + Treg), myeloid cells (Neutrophils, Alveolar M, cDC2/moDCs, Lipid-associated M, pDCs), B cells (B cell, Plasma, B activated), malignant epithelial cells (SOX2 Cancer, CXCL1 Cancer, CDKN2A Cancer, Proliferating Cancer), normal epithelial cells (Alveolar, Ciliated), fibroblasts (SMC, CAF), other immune cell types (NK, Mast), and stressed/unknown cells (Figures 3D,E). We examined the proportional distribution of cell subpopulations and found a decrease in the proportion of CD8 T cells, alveolar macrophages (Alveolar M), and natural killer (NK) cells in lung cancer patients. Conversely, there was an enrichment of T and B lymphocytes, suggesting activation of the adaptive immune response, alongside a decrease in NK and myeloid cells compared to normal lung tissue (Figure 3F). These findings indicate that the relationship between lung cancer and cytotoxic immune cell types can be elucidated at the single-cell level.

### 3.4 Gene expression and interactions at the single-cell level

**FIGURE 4:**
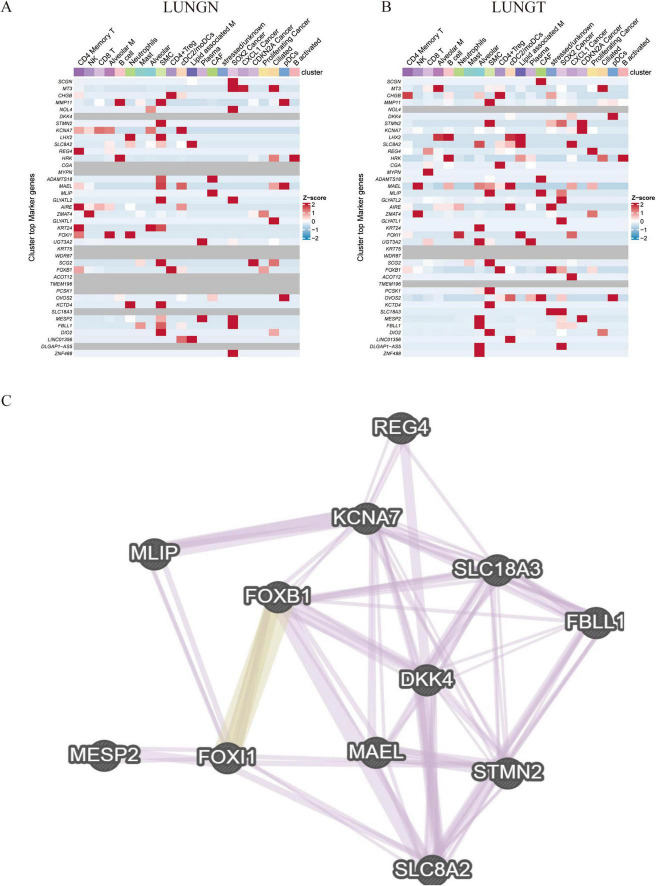
Validation of differentially upregulated gene expression at the single-cell level. **(A,B)** Thermogram: shows the expression of 40 differentially regulated genes in adjacent and cancer cell types. **(C)** Gene interaction network: displays a network of differentially regulated genes generated by Genemania.

In our single-cell analysis, we confirmed that the previously identified up-regulation of gene expression associated with metastatic recurrence comprised 40 up-regulated genes versus 509 down-regulated gene expression profiles. Notably, we identified 13 positively associated genes relevant to cancer, including DKK4, SLC8A2, MAEL, FOXI1, FBLL1, KCNA7, MESP2, STMN2, REG4, and FOXB1 (Figures 4A,B). To elucidate the functional roles of these core genes in protein interactions, we constructed a protein-protein interaction (PPI) network using the GeneMANIA database (Figure 4C). The results demonstrated significant co-expression relationships, indicating that these genes may play critical roles in pathways associated with cell proliferation and differentiation, such as DNA-binding transcription factor activity, RNA polymerase II specificity, sequence-specific DNA binding, myofilament assembly, and double-stranded DNA binding in the context of lung cancer recurrence.

### 3.5 Key gene validation

**FIGURE 5:**
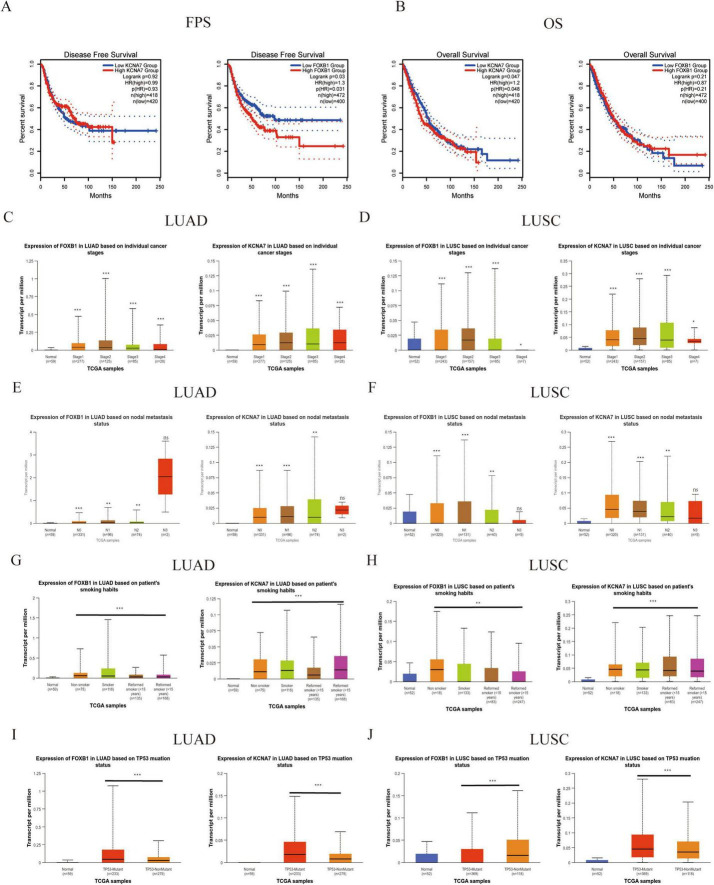
Prognostic significance of KCNA7 and FOXB1 in NSCLC. **(A)** KCNA7/FOXB1 and FPS: correlation with FPS in NSCLC. **(B)** KCNA7/FOXB1 and OS: correlation with overall survival (OS) in NSCLC. **(C,D)** KCNA7 mRNA and stage: relationship with cancer staging in NSCLC. **(E,F)** KCNA7 mRNA and LN metastasis: association with lymph node metastasis in NSCLC. **(G,H)** KCNA7 mRNA and smoking: correlation with smoking status in NSCLC. **(I,J)** KCNA7 mRNA and TP53 mut: relationship with TP53 mutation status in NSCLC. *Indicates a statistically significant difference at *p* < 0.05. **Indicates a highly significant difference at *p* < 0.01. ***Indicates an extremely significant difference at *p* < 0.001.

**FIGURE 6:**
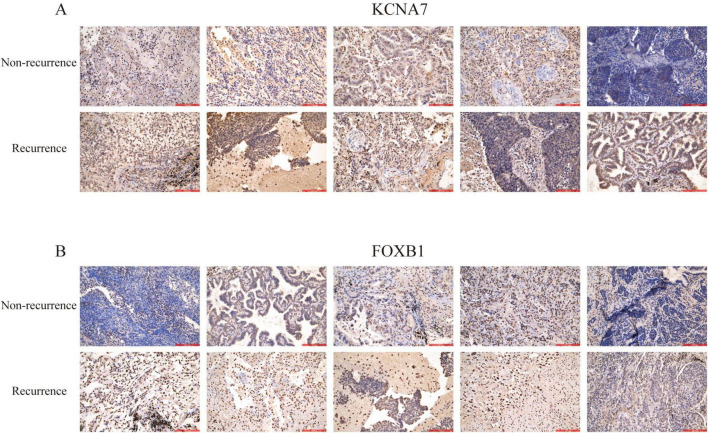
Immunohistochemical staining of lung cancer tissues. Representative images of lung cancer tissue samples showing the expression levels of key proteins. The upper layer non-recurrence tissues, while the lower layer shows recurrence tissues. Staining was performed using antibodies against **(A)** KCNA7 and **(B)** FOXB1.

To evaluate the potential of pivotal genes in predicting survival outcomes for lung cancer patients, we analyzed survival curves based on TCGA data. Among the 13 predicted hub genes, we observed statistically significant differences in progression-free survival (PFS) and overall survival (OS) for KCNA7 and FOXB1 in NSCLC patients compared to normal lung tissue (*P* < 0.05) (Figures 5A,B). Further analysis using the GEPIA2 database confirmed that the expression of KCNA7 and FOXB1 was significantly correlated with clinical survival in LUAD and LUSC patients compared to normal tissues (Figures 5A,B). Consequently, we propose that KCNA7 and FOXB1 function as core genes regulating lung cancer recurrence and are associated with poor survival outcomes in NSCLC patients (Figures 5C,D). We further explored the effects of KCNA7 and FOXB1 on clinical traits in lung cancer patients (Figures 5E,F). Our findings indicate that the expression levels of FOXB1 and KCNA7 increase with the progression of tumor cells, with the highest expression observed in patients who smoked and those with TP53 mutations (Figures 5G–J). To validate this hypothesis, we conducted immunohistochemical analysis on lung cancer patients with and without recurrence. The results revealed that the expression levels of KCNA7 and FOXB1 were significantly higher in patients with recurrence lung cancer compared to those in the non-recurrence group (Figure 6A,B). Collectively, these results suggest that FOXB1 and KCNA7 may serve as biomarkers for prognostic evaluation in lung cancer and provide a novel theoretical foundation for clinical treatment strategies.

## 4 Discussion

This study investigates gene expression differences and key pathways that influence primary and recurrent lung cancer states. In lung adenocarcinoma (LUAD), we identified 1,151 genes with upregulated expression and 1,088 genes with downregulated expression; in lung squamous cell carcinoma (LUSC), 735 upregulated and 2,669 downregulated genes were identified.

Using Gene Set Variation Analysis (GSVA) pathway enrichment analysis, we confirmed a strong connection between LUAD and LUSC recurrence, particularly concerning core biological pathways such as the cell cycle, including the E2F and G2M signaling pathways. The E2F family of genes, especially E2F1 and E2F2, exhibits elevated expression levels significantly associated with poor prognosis in patients with non-small cell lung cancer (NSCLC) (20). Notably, E2F1 is closely related to the well-known tumor suppressor gene RB1 (21). Under normal conditions, RB1 inhibits cell cycle progression, preventing cells from prematurely entering the S phase (DNA synthesis) and subsequent phases (22). However, RB1 inactivation lifts this inhibitory effect, allowing downstream genes critical for S phase entry and progression to be expressed (23).

Notably, several recognized oncogenes play a crucial role in RB1 inactivation mechanisms. For instance, cyclin-dependent kinase 4 (CDK4) and cyclin D1 can bind to and phosphorylate RB1, thereby neutralizing its cell cycle inhibitory effect (24, 25). This mechanism of RB1 inactivation has been observed across various cancers and is considered a key driver of cancer progression (26). Consequently, the high expression of E2F1 and E2F2, alongside RB1 inactivation and activation of downstream oncogenes, constitutes a complex regulatory network that significantly influences the onset, progression, and prognosis of lung cancer, particularly NSCLC. Targeted therapeutic strategies aimed at these critical nodes, such as inhibiting CDK4 or cyclin D1 activity or restoring RB1 function, are expected to offer new therapeutic avenues for lung cancer patients (27, 28).

The aim of this study was to identify genes associated with NSCLC recurrence. Using weighted gene co-expression network analysis (WGCNA) and single-cell techniques, we identified 40 candidate genes. Single-cell mapping revealed complex interactions between cancer subpopulations and specific genes, showing significant alterations in the proportions of CXCL1 and SOX2 cancer subpopulations. This suggests their direct involvement in lung cancer development and progression. Furthermore, nine key differential genes (SLC18A3, MLIP, OVOS2, DKK4, SLC8A2, MAEL, FOXI1, FBLL1, KCNA7) were identified in the two subpopulations. Survival analysis indicated that high expression levels of KCNA7 and FOXB1 correlate with poor prognosis in NSCLC patients. Thus, KCNA7 and FOXB1 emerge as critical genes for NSCLC prognosis and potential therapeutic targets.

As a member of the FOXO family of transcription factors, FOXB1 plays a pivotal role in tumorigenesis and is involved in various cellular physiological processes, including apoptosis, cell cycle control, glucose metabolism, and oxidative stress resistance through gene expression regulation (29, 30). Additionally, we discovered that FOXB1 co-regulates cystic fibrosis transmembrane conductance regulator (CFTR) expression with its family member FOXI1 (31). In lung cancer, only the clustered cell-like subpopulation significantly expresses FOXI1, which serves as a major regulator of ionocytes and a primary source of CFTR activity (32, 33). Based on these findings, we hypothesize that FOXI1 and CFTR are not only major regulators of lung ionocytes but also closely associate with CXCL1 cancer subpopulations. This relationship may elucidate a specific role for CXCL1 in lung carcinogenesis and progression, alongside the potential regulatory mechanisms of FOXB1 and FOXI1.

Potassium voltage-gated channel subfamily A member 7 (KCNA7), belonging to a group of potassium channel-associated genes, has garnered increasing attention in cancer research. Ion channels, particularly potassium channels, are extensively studied in cancer contexts due to evidence suggesting they may serve as effective targets for tumor therapy. Compared to normal cells, potassium channels are typically overexpressed in cancer cells, primarily due to their ability to enhance cancer cell proliferation by regulating cell membrane potential, calcium homeostasis, and multiple signaling pathways (34, 35). While other potassium voltage-gated genes such as KCNA2, KCNA3, and KCNA5 have been investigated across various cancers—including skin melanoma, uterine corpus endometrial carcinoma, gastric adenocarcinoma, LUAD, and LUSC in-depth studies of KCNA7’s expression in cancer remain lacking (34). It has been shown that the distribution of a single nucleotide polymorphism (cSNP) locus (e.g., T418M) of KCNA7 in the normal population adheres to Hardy-Weinberg equilibrium, with no significant difference between the gene frequency and genotype frequency of the locus in patients with non-insulin-dependent diabetes mellitus and healthy controls (36). However, this does not imply that KCNA7 is unrelated to cancer; rather, more specific investigations are needed to explore its expression and role in cancer. Notably, we identified for the first time an association between KCNA7 and the occurrence and progression of lung cancer. This finding highlights KCNA7’s potential role in lung cancer and provides new perspectives and targets for future treatments. Through further investigation into KCNA7’s functions and regulatory mechanisms, we aim to establish more precise and effective treatment strategies for lung cancer patients.

In summary, we propose a relationship between lung cancer recurrence and specific genes by integrating data from The Cancer Genome Atlas (TCGA) for squamous lung cancer and lung adenocarcinoma. Validation with single-cell data revealed distinct biological roles among different cancer subpopulations based on known cellular distributions. Our findings identify an important link to lung cancer development and highlight KCNA7, FOXI1, and FOXB1 as marker genes for CXCL1 cancer subgroups relevant to clinical prognosis.

## 5 Conclusion

This study utilized bioinformatics analyses to screen differentially expressed genes associated with recurrence, subsequently identifying key genes linked to proliferation via enrichment analysis. Following transcriptomic and single-cell histological analyses, we established that the highly expressed transcription factors FOXI1, FOXB1, and KCNA7 promote lung cancer development and are primarily involved in cellular processes including proliferation, migration, and invasion. These findings provide new molecular targets for addressing lung cancer recurrence.

## Data Availability

The datasets presented in this study can be found in online repositories. The names of the repository/repositories and accession number(s) can be found in the article/Supplementary material.

## Footnotes

1
http://gepia2.cancer-pku.cn/

2
https://ualcan.path.uab.edu/
